# Promotion of immune and glycaemic functions in streptozotocin-induced diabetic rats treated with un-denatured camel milk whey proteins

**DOI:** 10.1186/1743-7075-11-31

**Published:** 2014-07-01

**Authors:** Hossam Ebaid

**Affiliations:** 1 Department of Zoology, College of Science, King Saud University, Saudi Arabia, Riyadh, KSA; 2 Department of Zoology, Faculty of Science, El-Minia University, El-Minia, Egypt

**Keywords:** Lymphocyte proliferation, T and B cells, Pancreatic ß cells, Type 1 diabetes-rat model, Whey protein

## Abstract

T cell mediated autoimmune diabetes is characterized by immune cell infiltration of pancreatic islets and destruction of insulin-producing β-cells. This study was designed to assess the effect of whey proteins (WP) on the responsiveness of lymphocytes in rats after four months of Streptozotocin (STZ)-induced Type 1 diabetes (T1D). A diabetic group was supplemented with WP daily for five weeks at a dose of 100 mg/kg. Ribonucleic acid (RNA) was extracted from stimulated lymphocytes in order to analyse gene expressions using real time PCR and RT-PCR. PCR results were confirmed with ELISA. The proliferation capacity of lymphocytes and their homing to the spleen were studied. Antigen-activated lymphocytes showed that diabetes impaired the mRNA expression of the protein kinase B (Akt1), Cdc42, and the co-stimulatory molecule, CD28, which are important for cell survival, actin polymerization and T cell activation, respectively. Accordingly, proliferation of lymphocytes was found to be suppressed in diabetic rats, both in *vivo* and in *vitro*. WP was found to restore Akt1, Cdc42 and CD28 mRNA expression during diabetes to normal levels. WP, therefore, served to activate the proliferation of B lymphocytes in diabetic rats both in *vivo* and in *vitro*. Although WP was found to up-regulate mRNA expression of both interleukin (IL)-2 and interferon gamma (IFN-γ), it suppressed the proliferation activity of almost all T cell subsets. This was confirmed by WP normalizing the structure and function of ß cells. Meanwhile, WP was found to down regulate the mRNA expression of Tumor necrosis factor-alpha (TNF-α) and its programmed cell death-receptor (Fas). Taken together, the results of this study provide evidence for the potential impact of WP in the treatment of immune impairment in T1D, suggesting that it serves to reverse autoimmunity by suppressing autoreactive T cells and down regulating TNF-α and Fas, resulting in improved pancreatic ß cell structure and function.

## Introduction

Homeostasis within the immune system needs to selectively force the survival of useful lymphocytes in the central lymphoid organs and antigen-reactive cells in the periphery, whilst deleting strongly autoreactive cells in the thymus and bone marrow [1]. CD4+ T-helper cells differentiate into three subsets of effector cells: Th1, Th2, and Th17. An overactive Th1 response can lead to autoimmune diseases such as T1D [2]. T cell activation involves multiple interactions with the cell surface, including co-stimulatory molecules such as CD28, and signaling from cytokine or chemokine receptors [3]. Recent studies provide evidence that CD28 co-stimulation of different cytokines is mediated by discrete signaling pathways, one of which includes protein kinase B (Akt) [4] which is known to protect cells against apoptosis [5]. Dysregulation of Akt leads to diseases such as cancer, and diabetes as well as cardiovascular and neurological diseases [6] and schizophrenia [7]. Cdc42, a member of the Rho family of GTPases, plays a role in cell motility and migration [8,9] and regulates the dynamics of the cytoskeleton [10]. Runne et al. [11] found that Cdc42 activation regulates lymphocyte chemotaxis. Guo et al. meanwhile, [12] showed that Cdc42 maintains naïve T cell homeostasis through promotion of cell survival and suppression of T cell activation.

ß-cell apoptosis involves multiple signaling cascades stimulated by interleukin (IL)-1β, interferon-gamma (IFN-γ), and tumor necrosis factor-alpha (TNF-α) [13]. The TNF superfamily causes recruitment of several intracellular adaptors, such as Fas, to activate multiple signal transduction pathways that induce apoptosis [14]. The eventual fate of the cell is dependent on integrated signals received through the antigen receptor, co-stimulatory receptors, cytokine receptors and members of the TNF receptor family, with these signals being highly specialized to promote either the survival or death of the cell, and thus eventually to return the immune response to a state of homeostasis.

Identifying proteins that normally regulate immune response and decrease apoptosis in diabetics offers the opportunity to develop novel therapeutic strategies. Whey proteins (WP) has been postulated to reduce the effects of oxygen radicals by increasing glutathione [15-18], which stimulates lymphocyte proliferation, increases mast cells and their associated cytokines and biochemical mediators, and enhances the humoral immune response. Furthermore, a recent study has successfully determined the role of WP in restoring the normal inflammatory phase of the wound healing process in diabetic models [15] and the proliferation of PBMC [16]. WP is easily available from different milk sources, especially camels, in Saudi Arabia. The current study builds on this work by assessing whether WP can influence the outcome and progression of diabetic immune defects. Specifically, we investigated the impact of WP on ß cell functions and on lymphocyte activation and proliferation in an animal model of T1D.

## Materials and methods

### Preparation of un-denatured camel milk whey proteins

The milk was skimmed by centrifugation at 5000 g for 20 min using an IEC Model K centrifuge [Boston, USA]. Skim milk was acidified to pH 4.3 using 1M of HCl. The precipitated casein was removed by centrifugation, and the supernatant containing the whey protein was saturated with ammonium sulfate (70% saturation) and incubated overnight at 4°C. The precipitated whey proteins were collected by centrifugation and dialyzed against distilled water for 48 h at 4°C using a Spectra/Pro® Membrane, MWCO 6000-8000 Da. The obtained dialyzate was lyophilized using a Unitop 600 SL, [Virtis Company, Gardiner, New York 12525 USA] and were kept at -20°C until use. The dialyzate containing un-denatured whey proteins were freeze-dried and refrigerated until use.

### Ethical approval

Camel milk was obtained from a camel breed (Majaheem) from the Najd region (Alazeria farm; GPS: 300 02 47/ 300 02 27) in Saudi Arabia. Specific permissions were not required for activities in this private farm. This study did not involve endangered or protected species. Regarding experimental animals, all procedures were conducted in accordance with the standards set forth in the guidelines for the care and use of experimental animals by the Committee for the Purpose of Control and Supervision of Experiments on Animals and the National Institutes of Health. The study protocol (care and handling of experimental animals) was approved by the Animal Ethics Committee of the Zoology Department in the College of Science at King Saud University.

### Diabetic models

Diabetes was induced by a single injection of freshly dissolved STZ (60 mg/kg of body weight; Sigma, USA) in a 0.1 mol/l citrate buffer (pH 4.5) into the peritoneum [19]. Control rats were injected with citrate buffer. Seven days after STZ injection, the rats were screened for serum glucose levels. Rats with a serum glucose level ≥ 200 mg/dl after 2 h of glucose intake were considered diabetic and selected for further studies.

### Experimental diet

Rats were supplemented with whey protein in the diet as previously described [20-22]. To prepare 500 g of the diet, 5 g vitamins, 25 g mineral salts, 40 g fats, 50 g sucrose, 100 g protein (20% protein) and 280 g starch were mixed. Casein was the protein source in both the control and the diabetic groups. The un-denatured camel milk whey protein was the protein source in the WP-treated diabetic group. The diet was kept at 4°C until use [23].

### Experimental design

A total of 45 male rats (12-week-old), weighing 120-150 g each, were obtained from the Central Animal House of the Faculty of Pharmacy at King Saud University. All animals were allowed to acclimatize in metal cages inside a well-ventilated room for 2 weeks prior to the experiment. Animals were maintained under standard laboratory conditions (temperature at 23°C, relative humidity was 60%–70% and a 12-h light/dark cycle) and were fed a diet of standard commercial pellets and given water ad libitum. Animals were distributed into three experimental groups (n = 15/group): group I was daily administered 1% carboxymethyl cellulose (CMC), group II diabetic rats (DM) was supplemented with distilled water (250 μl/rat/day) for five weeks and group III was supplemented with camel milk un-denatured WP (DMWP) (100 mg/kg/body weight dissolved in 250 μl/day) for five weeks. Rats of the third group were freely supplemented with camel milk un-denatured whey proteins as a protein constituent of the diet. An additional supplementary group of normal rats treated with WP was studied for confirming the results of the three main groups. However, data from these groups are not included in this study.

### Collection of samples

The animals from all groups were autopsied under light ether anesthesia. At the end of the experimental period, blood was drawn from the animals by puncturing retro-orbital venous sinus with capillary tubes until killing. Sera were used for the determination of glucose level. After collection of blood samples, the animals from all groups were autopsied under light ether anesthesia. Subsequently, spleen was excised from surrounding tissues and parts were placed into tubes with fixatives (10% formalin) for histological and immunochemical studies. Other splenic parts were placed in buffers for lymphocytes isolation.

### Estimation of glucose and insulin concentration

Serum glucose concentration was determined according to the Trinder method [24] using a commercial diagnostic kit (Biodiagnostics, Egypt). Serum insulin was assayed using a DPC radioimmunoassay kit (Diagnostic Products Corporation, Los Angeles, USA) [coat-A-count] according to the method reported by Marschner et al. [25].

### Lymphocyte isolation

Rats were anesthetized and euthanized; spleen was taken out immediately and rinsed with cold phosphate buffered saline and then placed on a 200-mesh stain steel sieve and grounded with plunger of glass syringe. Two volume of isolated lymphocyte separation medium was added, mixed gently, and centrifuged at 2,000 × g for 10 min. The top layer was discharged leaving the middle lamella layer, showing milk cream color, contained lymphocytes. Five volume of PBS buffer was added and centrifuged at 1,000 × g for 5 min. Precipitated lymphocytes were re-suspended in RPMI-1640 medium (making the final concentration of 1 × 10^6^ cells/mL). The viability of lymphocytes was analyzed using trypan blue, and in all acceptable preparations, it exceeded 95%.

### T and B lymphocyte proliferation assay

The proliferation was determined in sterile flat-bottomed 96-well culture plate. Lymphocyte suspension was on the plate, then RPMI-1640 medium containing concanavalin A (Con A, making the final concentration of 5 mg/L for T cell or 10 mg/L lipopolysaccharide for B cells) was added and incubated at 37°C under 5% CO_2_ for 24 h. Then, 20 μL MTT (5 g/L) was added to each plate and incubated for another 4 h. The supernatants were sucked and discarded; 150 μL of dimethyl sulfoxide was added to each plate and shaken. The optical absorbance at 490 nm was recorded in an ELISA plate reader as previously described [26,27]. There were ten replicates for each rat lymphocytes.

### Immunohistochemical detection of proliferated cell nuclear antigen (PCNA), T and B cells

Spleen samples were fixed in 10% neutral buffered formalin Paraffin sections were cleared in xylene, rehydrated in graded ethanol (100%-70%), immersed in water for 5 to 10 minutes, and incubated in 0.3% H_2_O_2_ in 70% methanol for 20 minutes to inhibit endogenous peroxidase activity. The specimens were then rinsed three times for 5 minutes in PBS, and epitopes were unmasked by boiling in citrate buffer (pH 6.0) for 15 minutes, when necessary. The sections then were blocked for 60 minutes in 3% BSA in PBS or in 1% normal goat serum and 3% non-fat milk. Section were incubated with primary antibodies (Anti-PCNA [Novo Castra NCL-PCNA], anti-CD3; pan T cells or anti-CD20; pan B cells) in 0.1% BSA overnight at 4°C in a humidified chamber. Samples were then rinsed in PBS and incubated with biotinylated secondary antibody in 0.1% BSA for 1 hour at room temperature, followed by avidin biotin amplification (ABC Elite) for 30 minutes. Sections were developed with 3,3-diaminobenzidine peroxidase substrate (Sigma). Sections were counterstained with Mayer haematoxylin for 3 minutes and mounted. Negative controls were set by substituting the primary antibody with PBS [28]. Photographs of the sections were taken; the images were digitized using Adobe Photoshop (Adobe Systems, Mountain View, CA). The PCNA (the number of brown stained cells), anti-CD3 or anti-CD20-stained cells were determined at 20 random locations within the spleen follicles and the PALS for each animal from each group using a Leica Qwin 500 image analyzer.

### RNA extraction and RT-PCR

RNA was extracted from the collected samples (RNA latter) using RNeasy Mini Kit (QIAGEN) according to the manufacturer instructions. RT-PCR was performed using QIAGEN One Step RT-PCR kit as directed by the manufacturer’s instruction manual. The desired genes were amplified using specific primers (e-oligos, Hawthorne, USA) listed in Table 1. For each sample, 25 μl reaction mixture was performed. PCR reaction was carried out using Gene-Amp 9700 thermal cycler. RT-PCR products were analyzed in 1.2 % agarose gel. PCR conditions were optimized to allow semiquantitative comparisons of results, as previously described [29]. The levels of the three mRNA and β-actin mRNA were quantified by gel electrophoresis and densitometry. mRNA levels were normalized versus β-actin and are expressed in arbitrary units.

**Table 1:** List of primers used to amplify the desired genes

**Primer name**	**5′——————— < 3′**		**Primer length**
IL-2	Forward	CCTGAGCAGGGAGAATTACA	20
	Reverse	TCCAGAACATGCCGCAGA	18
Fas	Forward	GAGAATTGCTGAAGACATGACAATCC	26
	Reverse	ATGGCTGGAACTGAGGTAGTTTTCAC	26
Akt1	Forward	CCTTTATTGGCTACAAGGAACGG	23
	Reverse	GAAGGTGCGCTCAATGACTG	20
CD28	Forward	GTATTCCTACAACCTTCTCGCAA	23
	Reverse	GGGGCTGATAGGTAAAATTCCCA	23
Cdc42	Forward	TTCTGGTTGTGTTTCAACTGCT	22
	Reverse	CCTCCCTTGGACTGCATCTG	20
Inf- γ	Forward	GCCCAATATCTCGGATGCTTC	21
	Reverse	GCCAAAATAGCTTCGGTAATCCT	23
TNF-α	Forward	CCAACATGCTGATTGATGACACC	23
	Reverse	GAGAATGCCAATTTTGATTGCCA	23

Quantification of mRNA expression by real-time polymerase chain reaction cDNA from the above preparation was subjected to PCR amplification using 96-well optical reaction plates in the ABI Prism 7500 System (Applied Biosystems®). The 25-μl reaction mixture contained 0.1 μl of 10 μM forward primer and 0.1 μl of 10 μM reverse primer (40 μM final concentration of each primer), 12.5 μl of SYBR Green Universal Mastermix, 11.05 μl of nuclease-free water, and 1.25 μl of cDNA sample. The primers used in the current study were chosen from pubmed.com. The RT-PCR data was analyzed using the relative gene expression method, as described in Applied Biosystems® User Bulletin No. 2. The data are presented as the fold change in gene expression normalized to the endogenous reference gene and relative to a calibrator.

### Statistical analysis

The statistical analysis was performed using the MINITAB software (MINITAB, State College, PA, Version 13.1, 2002). The data from the experiments were tested for normality using the Anderson Darling test, and for variance homogeneity prior to any further statistical analysis. The data were normally distributed with homogeneous variances. Thus, the one-way ANOVA statistical measure was used to determine the overall effect of each treatment. This measure was supplemented by individual comparison between the different treatments using Tukey’s method for pairwise comparisons. The results were expressed as arithmetic mean (M) ± standard deviation (SD). Only statistically significant differences with P < 0.05 were found between the treatment group and the control, and between the treatment group and the diabetic group considered.

## Results

### WP restores Akt1, Cdc42 and CD28 signaling during diabetes

**Figure 1:**
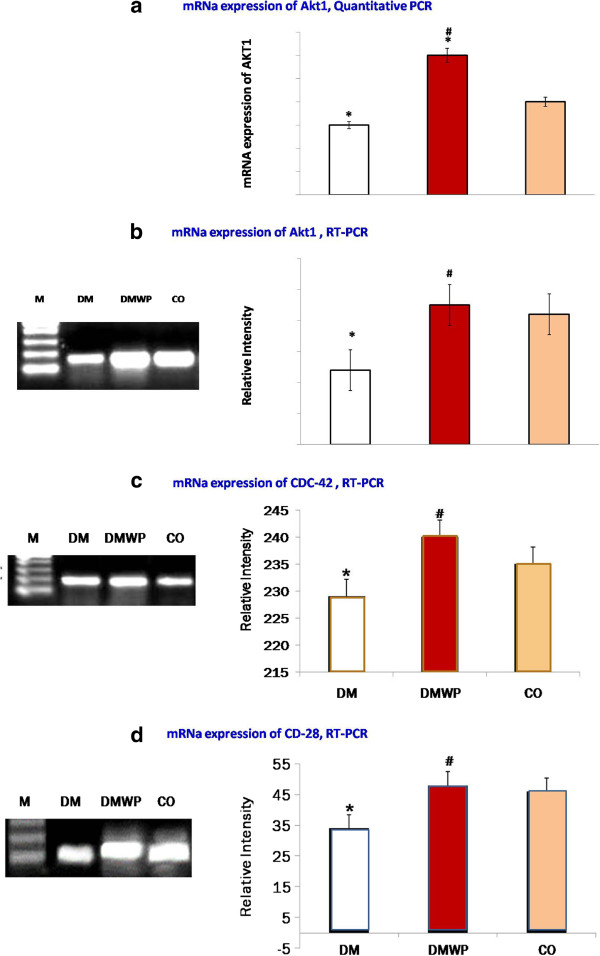
**Quantitative analysis of mRNA expression of AKT1 genes (a).** Semiquantative PCR-mRNA expression of Akt1 **(b)**, CD28 **(c)** and Cdc42 **(d)** of the antigen activated lymphocytes from control, DM and DMWP rats. PCR products were separated and visualized by DNA agarose electrophoresis after amplification of Akt1, CD28 and Cdc42 from splenocyte genomic DNA. Samples from five animals were analyzed. A representative result from each group is presented, while the values in the histograms are the mean ± SD. *shows the significance (*p* < 0.05) in comparison to the control group. #shows the significance (*p* < 0.05) in comparison to the diabetic group.

To assess whether WP supplementation could influence T-cell survival, activation and proliferation in T1D, we investigated the mRNA expression of Akt1, CD28 and Cdc42. mRNA expression of the activated lymphocytes showed that diabetes impaired the Akt1 (234 ± 9.5), CD28 (229 ± 14) and Cdc42 (33 ± 6.5) signaling compared to those of the control group [Akt1 (252 ± 12), CD28 (235 ± 10), Cdc42 (45 ± 5.0)]. WP supplementation, however, was found to restore mRNA expression of Akt1 (255 ± 16), CD28 (240 ± 14) and Cdc42 (47 ± 4.0) close to the normal level (Figure 1a,b,c,d). Up-regulation of Akt1, Cdc42 and CD28 expression was also evident in diabetic rats supplemented with WP.

### WP activates lymphocyte proliferation in diabetic rats in *vivo* and in *vitro*

**Figure 2:**
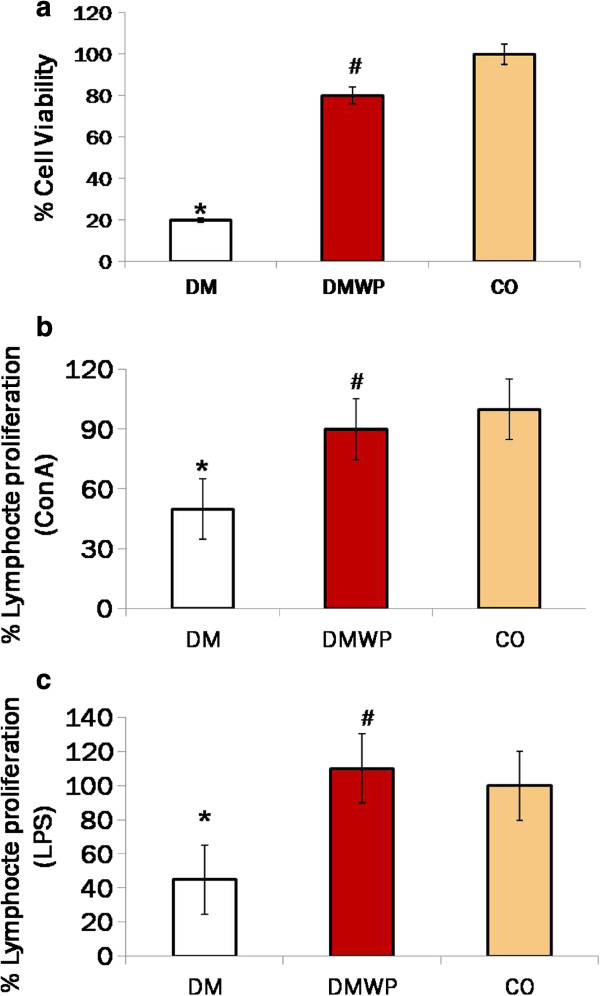
**MTT assay for the cell viability percentage of control splenocytes (a).** Splenic lymphocytes were co-cultured with mitogen [suspension containing con. A for detecting activity of T cell **(b)** or LPS for detecting activity of B cells **(c)**] for 48 h and then harvested for analysis with the MTT method.

**Figure 3:**
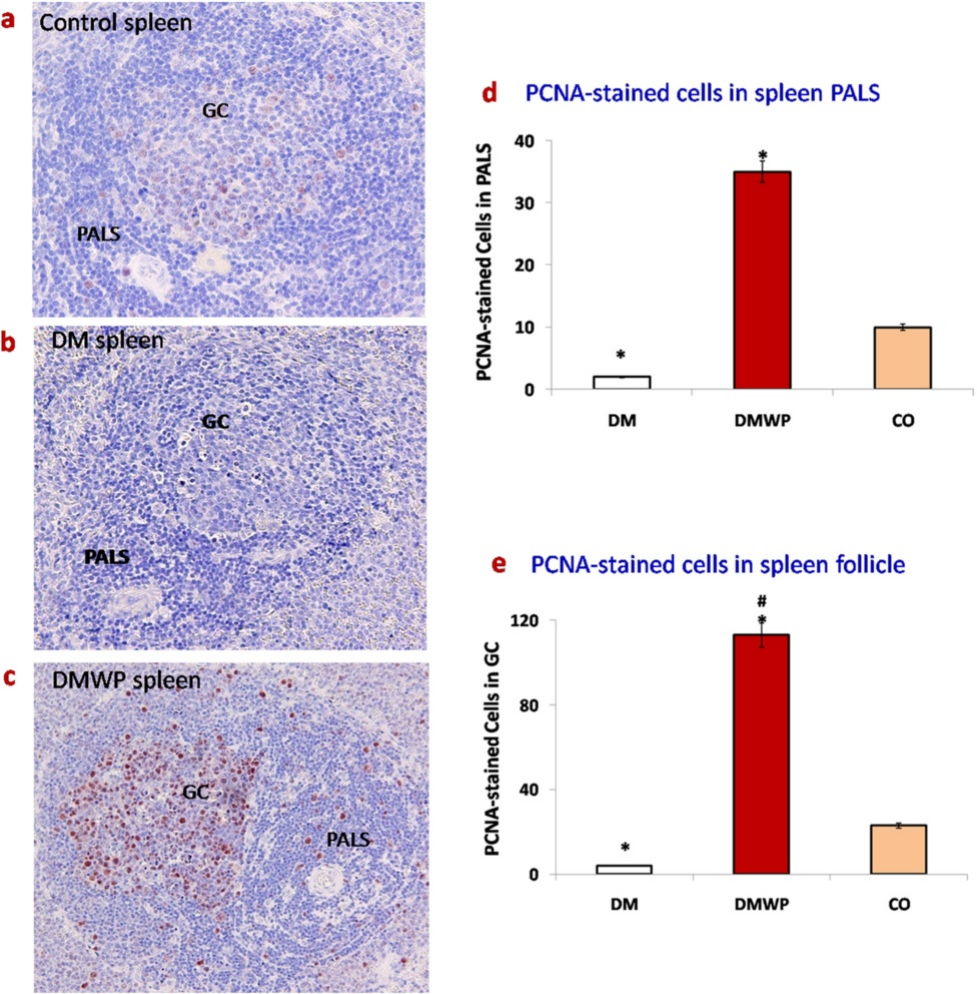
**Spleen sections were stained with PCNA antibodies (×400) to show proliferation activity in control (a), DM (b), DMWP (c) spleens showing the brown proliferating cells in the PALS and germinal centers (GC).** Values shown are the mean count of the proliferating cells in both PALS **(d)** and GC **(e)** ± SD. *shows the significance (*p* < 0.05) in comparison to the control group. #shows the significance (*p* < 0.05) in comparison to the diabetic group.

To validate these observations and to determine the effect of WP on lymphocyte activities, lymphocytes from the WP-treated diabetic rats, the diabetic and the control groups were isolated and examined for cell proliferation. Viability of the lymphocytes was measured by trypan blue exclusion test. Approximately 80% lymphocytes of diabetic rats were killed in medium during 72 hours of incubation comparing to the control rats (Figure 2a). Non-significant changes of the lymphocytes derived from WP-treated diabetic rats comparing to the control rats (Figure 2a). The mitogen stimulated (Con A or LPS) lymphocytes derived from WP-treated diabetic rats demonstrated significantly higher proliferative activity (90% or 110%, respectively) as compared to those from the diabetic rats (50% or 45%, respectively) (Figure 2b,c).To examine the impact of WP on lymphocyte proliferations further, spleen sections were stained with PCNA antibodies. PCNA-stained sections appeared to exhibit minimal proliferation activity in diabetic group compared to the control group (Figure 3). Lymphocytes stained with PCNA antibodies in the germinal centers of follicles from WP-treated rats showed very high proliferation activity (Figure 3). Periarteriol lymphatic sheath (PALS) cells (almost T cells) from WP-treated rats, showed a significantly higher capacity (35 ± 4.55) for proliferation than those of diabetic animals (2.0 ± 0.06) (Figure 3). In spleen follicle, cells from WP-treated rats, showed more than twenty eight fold higher capacity for proliferation (113 ± 7.45) than those of diabetic animals (4.0 ± 0.78) (Figure 3).

### WP forces survival of B lymphocytes and strongly suppresses T cells

**Figure 4:**
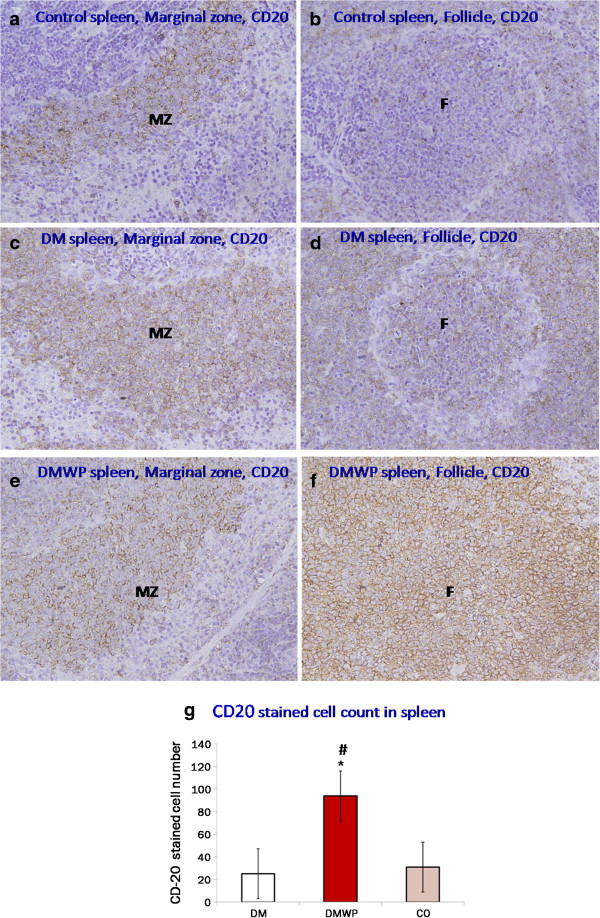
**Spleen sections stained with anti-CD20+ antibodies to realize B cells.** It shows the marginal zone and lymphatic follicles in the control **(a, b)**, DM **(c, d)** and DMWP **(e, f)** groups. The number of B cells was reduced in the marginal zone (MZ) and lymphatic follicles (F) of the diabetic rats and increased in both areas in WP treated diabetic rats (×400). Values shown are the mean count of the CD-20 cells in both MZ and F **(g)** ± SD. *shows the significance (*p* < 0.05) in comparison to the control group.

Following the observed mitogenic activity of WP shown previously in this work, we determined the extent to which both B and T cells were activated by this protein. Spleen sections were stained with anti-CD20 or anti-CD3 antibodies to realize B or T cells, respectively (Figure 4a-f). Figure 4g shows that the number of B cells was significantly (*P* < 0.05) reduced in both the marginal zone and lymphatic follicles of the diabetic rats (25 ± 1.5) compared to the control group (31 ± 1.4). On the other hand, the number of B cells in diabetic rats supplemented with WP were significantly (*P* < 0.05) increased (94 ± 4.59) in both the marginal zone and lymphatic follicles compared to the diabetic and the control rats, respectively (Figure 4g).

**Figure 5:**
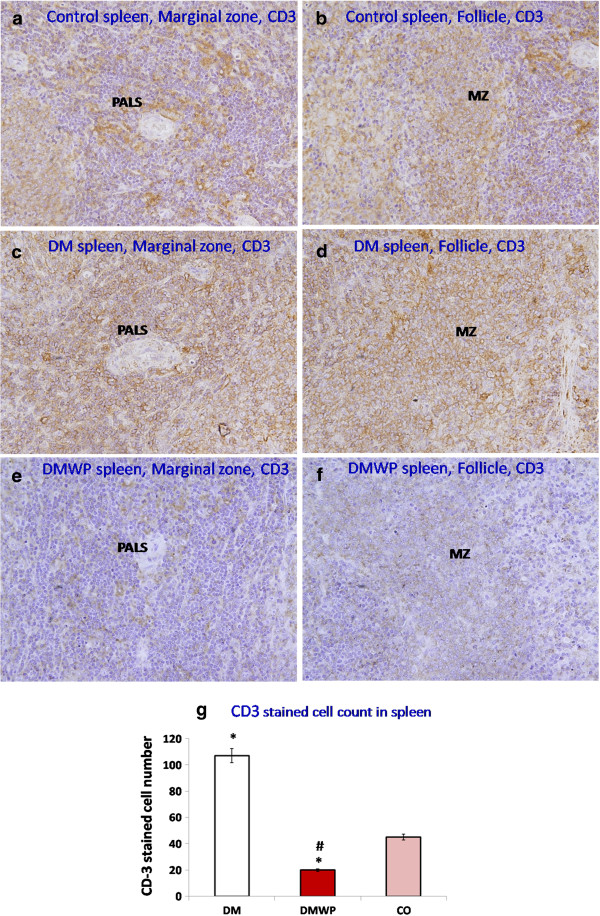
**Spleen sections stained with anti-CD3+ antibodies to realize T cells.** It shows the marginal zone and lymphatic follicles in the control **(a, b)**, DM **(c, d)** and DMWP **(e, f)** groups. T cells are strongly distributed in all zones, especially PALS, in the spleen sections of the diabetic rats. Interestingly, WP greatly reduced the number of T cells in the tissues of diabetic rats (×400). Values shown are the mean count of the CD-3+ cells in both MZ and F **(g)** ± SD. *shows the significance (*p* < 0.05) in comparison to the control group.

In addition, T cells, which destroy pancreatic ß cells, were extensively distributed in all zones, especially in the PALS, in the spleen sections of the diabetic rats (Figure 5c,d) (2.5-fold compared to the control group). WP was found to significantly (*P* < 0.05) reduce these T cells (20 ± 3.1) one fifth and one half their number in the diabetic (107 ± 11) and control (39 ± 3.6) groups, respectively (Figure 5g).

### WP increases polyfunctional T cells and down regulates mRNA expression of TNF-α and Fas during diabetes

**Figure 6:**
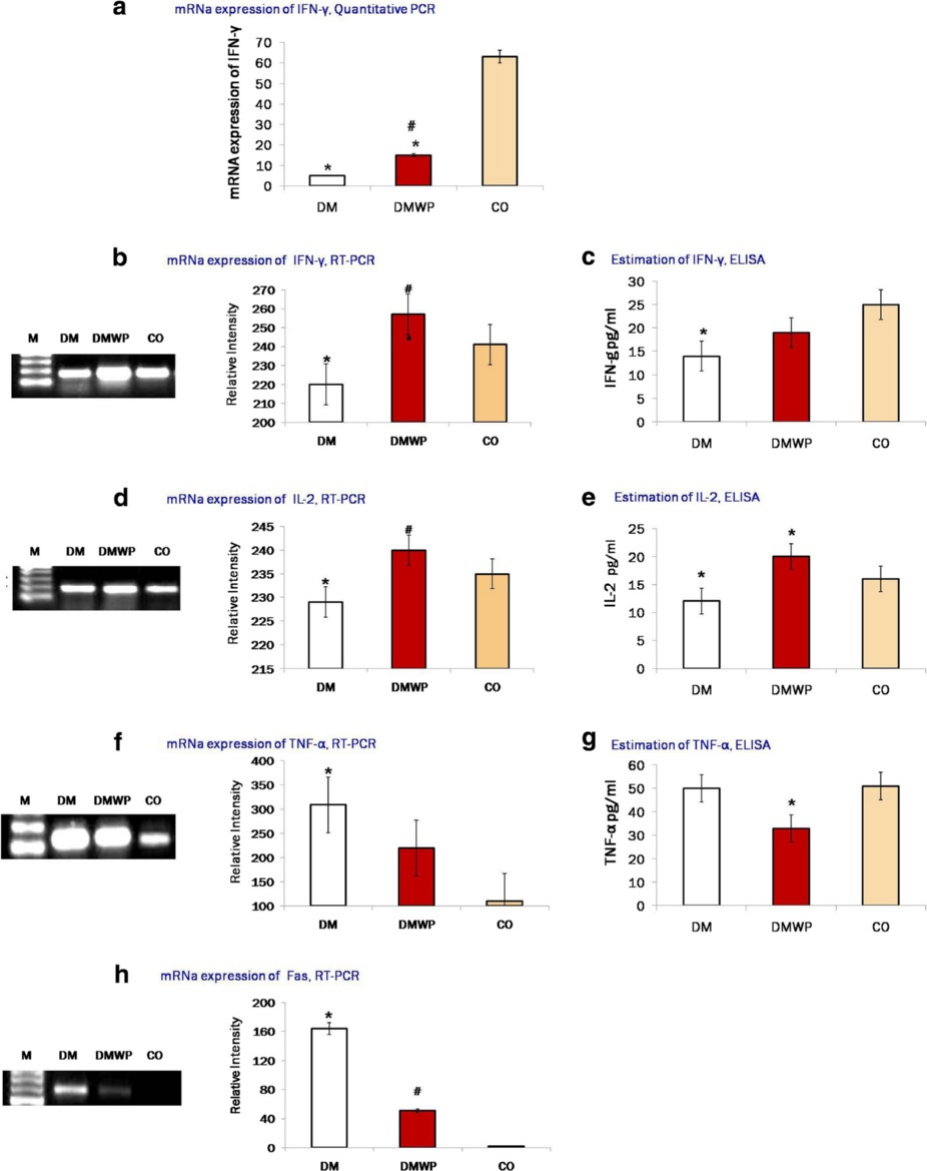
**Quantitative analysis of mRNA expression of AKT1 genes (a).** Semiquantative PCR-mRNA expression and the supernatant levels of IFN-γ **(b, c)** and IL-2 **(d, e)**. It was found that WP restores the polyfunctional T cells (IFN-γ and IL-2 producing cells that exhibit a high proliferation capacity). The mRNA expressions **(f)** and the supernatant level **(g)** of TNF-α, and the mRNA expressions of Fas **(h)**. Samples from five animals were analyzed. Samples from five animals were analyzed, while a representative result from each group is presented in this figure. Values shown are mean values ± SD. *shows the significance (*p* < 0.05) in comparison to the control group. #shows the significance (*p* < 0.05) in comparison to the diabetic group.

After exploring the impact of WP on the proliferative activity of both T and B cells the polyfunctional activity of T cells was investigated by detecting the expression of two important cytokines, IFN-γ and IL-2-mRNA. As shown in Figure 6a,b, mRNA expression of IFN-γ, which has a critical role in intracellular immune activity, was significantly (*P* < 0.05) impaired in the diabetic rats [4 (Figure 6a) and 2-fold (Figure 6b) reduced compared to the control group]. IFN-γ concentration detected by ELISA showed similar behavior in diabetic group (Figure 6c). IFN-γ mRNA in diabetic rats supplemented with WP were significantly (*P* < 0.05) 3-fold (15 ± 2.67) increased compared to the diabetic rats (5.0 ± 1.1) (Figure 6a).

In regard to the mRNA expression of IL-2, however, no significant difference was observed between the diabetic and control groups (Figure 6d) despite its protein level was significantly (*P* < 0.05) lowered (Figure 6e). Although WP minimized the number of T cells in the splenic tissues, WP was found to significantly restore the number of polyfunctional T cells (i.e. IFN-γ and IL-2-producing cells). WP was found to significantly (*P* < 0.05) elevate the mRNA expression of IL-2 (250 ± 12.7) compared to the diabetic (148 ± 6.8) and control (119 ± 4.9) groups (Figure 6d).To validate this data and to ascertain the impact of WP on the pancreatic ß-cell mass, we observed mRNA expression of TNF-α and one of its receptors (Fas), which, together with inflammatory cytokines, are the key factors in the destruction of ß-cell mass in T1D. The mRNA expression of TNF-α was significantly increased in diabetic rats (309 ± 14.55) but, in contrast, WP treatment was found to normalize the mRNA expressions of this gene (220 ± 9.11) (Figure 6f). By ELISA, WP was found to significantly reduce TNF-α (Figure 6g). The mRNA expression of Fas was significantly increased in diabetic rats (164 ± 7.29) compared to the control group (2.0 ± 0.034) (Figure 6h).

### WP normalizes the structure and function of pancreatic ß cells

**Figure 7:**
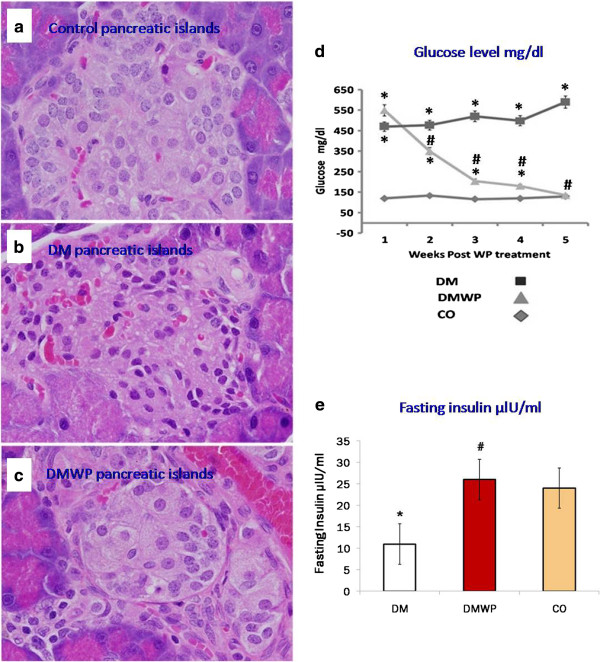
**Representative microscopic sections of the control (a), DM (b) and DMWP (c) rats (E&H × 400).** Plasma glucose **(d)** and insulin **(e)** concentration of the control, DM and DMWP rats. *shows the significance (*p* < 0.05) in comparison to the control group. #shows the significance (*p* < 0.05) in comparison to the diabetic group.

Furthermore, the histological structure of the pancreas from the three different groups was investigated to assess whether there were any observable impacts on the diabetic rates of supplementation with WP. While degenerative changes in the nuclei of the pancreatic ß-cells were observed in the microscopic sections of the untreated diabetic rats, with nuclei appearing darkly stained with fragmented chromatin (Figure 7b), in those animals treated with WP, more normal morphological features of the pancreatic ß-cells were restored (Figure 7c).

An improvement in the pancreatic cell function was also found. WP supplementation improved glucose clearance, enabling the basal plasma glucose concentration in those diabetic animals treated with WP to be significantly (*P* ≤ 0.005) normalized to the concentration levels in the control animals (Figure 7d). This confirmed that insulin release from the pancreatic islets was restored to normal levels (24 ± 2.6) in diabetic rats supplemented with WP (26 ± 2.28) (Figure 7e).

## Discussion

Inflammatory cytokines stimulate multiple signaling cascades leading to ß cell apoptosis [13] in T1D [29]. By suppressing inflammatory cytokines, WP increases the capacity of diabetic [15,17] animals to heal wounds. Potential effects of WP on immune processes, including the regulation of cytokines [30] and enhancement of the proliferation capability of PBMCs [16] have been observed. WP, therefore, can not only suppresses the inflammatory cytokines from autoreactive T cells but also restore normal ß-cell mass and function during diabetes.

In this study diabetes was found significantly to impair the proliferative response of splenic lymphocytes *in vivo* and antigen-stimulated lymphocytes *in vitro*. Indeed, we did not observe any significant proliferation activities in the PALS zone, which are provoked by CD4+ T cells. Similarly, Aarnisalo et al. [31] found a marked reduction in the proliferative response of CD4+ T cells among patients with T1D.

It is evident from our results that, in WP-treated rats, lymphocytes in splenic follicles showed highly significant proliferating activity, and this was confirmed in *vitro*. Although WP activates cell proliferation, therefore, staining with anti-PCNA antibodies showed that this proliferation was located in the B cell zone but not in the T cell zone of the splenic tissue. The proliferation activity stimulated by WP, therefore, was directed toward B cells rather than T cells. This finding was confirmed by staining with anti-CD20+ and anti-CD3+ antibodies which demonstrated that the number of B cells was greatly increased but that the number of T cells was reduced in the tissues of the WP-treated diabetic rats compared with untreated diabetic rats. This provides further evidence that WP is associated with B cell stimulation and T cell suppression during diabetes.

Our results suggest that WP may suppress the Th1 type of T cell that has a critical role in diabetic complications. This suggestion was practically confirmed by the up-regulation of Cdc42 expression, which maintains the homeostasis of naïve T cells by promoting cell survival and suppressing T cell activation [12]. Cdc42-deficient naïve T cells display impaired actin polymerization and show an enhanced differentiation to Th1 and CD8+ effector and memory cells [12]. Restoring Cdc42 by WP may, therefore, maintain the balance between the Th1 and Th2 subsets of T cell by specifically suppressing T cell activation and differentiation to Th1 and CD8^+^ effector and memory cells. Ultimately, this T cell homeostasis results in an improvement in the condition of the pancreatic ß cells.

Akt is critical for cell survival and is triggered by different stimuli [32]. WP supplementation was found to restore mRNA expression of Akt1 to the normal level. It was also shown in this work that up-regulation of Akt1 was accompanied by a significant increase of lymphocyte proliferation in the splenic tissues. The mRNA expression of the activated lymphocytes showed that diabetes had decreased the Akt1 signaling. Inhibition of cell migration has also been shown to be effected by inhibition of phosphorated PI3K/Akt, resulting in rapid Cdc42 proteolysis [33]. These studies confirm that Akt depletion impairs the mRNA expression of Cdc42 by activated lymphocytes during diabetes. Taken together, the results of our study confirm that WP linked up-regulation of Akt1 significantly elevates Cdc42 and this enhances T cell homeostasis but not ß cells attacking Th1 cells.

Cell death stimuli signals are either an intrinsic, mitochondrial pathway of apoptosis or can kill the cell through one of the six death receptors such as Fas [1]. Fas was significantly up-regulated in diabetic rats, as shown by the high number of dead lymphocytes in the diabetic group, suggesting that diabetic complications and oxidative stress induce cell apoptosis via Fas up-regulation [34,35]. The increased expression of Fas in the diabetic lymphocytes was, however, significantly inhibited by WP treatment. This indicates that WP restored T cell homeostasis by suppressing T cell proliferating activity rather than through the Fas-mediated apoptotic pathway.

Turning now to CD28, we found a lower level of CD28 expression on antigen-stimulated lymphocytes in the diabetic group while this level was restored in diabetic rats treated with WP. Recent studies have demonstrated that a significantly lower level of CD28 surface expression on T cells was detected in diabetic rats, children with T1D, cell leukaemia, chronic lymphocytic leukaemia and colorectal cancer [36-38]. CD28 may contribute to T cell viability by increasing glucose metabolism in activated T cells [39]. CD28 signals are also required to protect T cells from Fas-mediated apoptosis by activating the PI3K/Akt pathway [40]. Thus, a lower level of CD28 surface expression on T cells from diabetic rats could explain the observed two-fold higher level of dead cells in the diabetic group. In the absence of co-stimulation, cytokine secretion and T cell expansion, proliferation, survival and memory development are affected [41,42]. The diabetic rats in our study also exhibited decreased IL-2 or IFN-γ expression, which is a major T cell function. On the other hand, it is likely that the higher level of CD28 surface expression in the WP-treated animals causes the higher viability and proliferating capacity, and the restoration of IL-2 and IFN-γ levels.

WP suppresses TNF-α production, which is the key factor in ß cell-destruction in T1D since TNF-α controls the expression of the inflammatory gene network and contributes to the pathological complications observed in many inflammatory diseases such as schizophrenia and diabetes [43,44]. Therefore, while the higher expression of TNF-α caused pancreatic damage and dysfunction in the diabetic group in this study, WP was found to regulate expression of TNF-α and its apoptosis receptor, Fas to normal levels. By increasing glutathione, WP also induces oxidative stability leading to suppression of the inflammatory cascade [15]. Since blocking the production of TNF-α improves blood glucose concentrations, targeting TNF-α could effectively reduce expressions of the primary factors behind the complications associated with diabetes [45]. Thus, normal morphological features of the pancreatic ß-cells and glucose clearance were restored in the diabetic rats treated with WP due to the suppression of TNF-α. The hypoglycaemic effect of WP in individuals with T1D has recently been confirmed [46,47] and WP has also been shown to restore the capacity of the pancreatic islet to secrete insulin [48]. Here, we add to this picture of the beneficial effects of WP in the context of diabetes by showing that WP supplementation for five weeks in normalized glucose clearance in diabetic rats.

In conclusion, we have shown here that there is a strong, positive correlation between WP and immune function during diabetes in an animal model. WP restored the normal immune response as follows: 1) by activating the Akt1 pathway, it activated the CD28 signals required to protect T cells from Fas-mediated apoptosis; 2) by stimulating Cdc42, which maintains naïve T cell homeostasis by promoting cell survival and suppressing T cell activation. This leads to the restoration of the polyfunctional T cells (IFN-γ and IL-2 production), increased cell viability, and may restore the T cell subset balance; 3) by suppressing TNF-α and its receptor, Fas. The positive outcome of this range of effects at the cellular level are confirmed by our evidence on improvements of the pancreatic ß cell mass and function. There is a need, however, to investigate more intensively the potential role of WP in the treatment of diabetic immune impairment.

## Abbreviations

Cdc42: Cell division control protein 42; MTT: Cell proliferation assay; CD28: Co-stimulatory molecule; H&E: Haematoxylin-eosin; (IL)-2: Interleukin; IFN-γ: Interferon gamma; Fas: Programmed cell death-receptor; PBMC: Peripheral blood mononuclear cells; PCNA: Proliferated cell nuclear antigen; Akt1: Protein kinase B; TCR: T cell receptor; STZ: Streptozotocin; TNF-α: Tumour necrosis factor-alpha; T1D: Type 1 diabetes; WP: Whey protein.

## Competing interests

The author declares that there to be no competing interests.
